# Excimer Laser Phototherapeutic Keratectomy for the Treatment of Clinically Presumed Fungal Keratitis

**DOI:** 10.1155/2014/963287

**Published:** 2014-05-07

**Authors:** Liang-Mao Li, Li-Quan Zhao, Ling-Hui Qu, Peng Li

**Affiliations:** Department of Ophthalmology, No. 181 Hospital of PLA, Guilin 541002, China

## Abstract

This retrospective study was to evaluate treatment outcomes of excimer laser phototherapeutic keratectomy (PTK) for clinically presumed fungal keratitis. Forty-seven eyes of 47 consecutive patients underwent manual superficial debridement and PTK. All corneal lesions were located in the anterior stroma and were resistant to medication therapy for at least one week. Data were collected by a retrospective chart review with at least six months of follow-up data available. After PTK, infected corneal lesions were completely removed and the clinical symptoms resolved in 41 cases (87.2%). The mean ablation depth was 114.39 ± 45.51 **μ**m and diameter of ablation was 4.06 ± 1.07 mm. The mean time for healing of the epithelial defect was 8.8 ± 5.6 days. Thirty-four eyes (82.9%) showed an improvement in best spectacle-corrected visual acuity of two or more lines. PTK complications included mild to moderate corneal haze, hyperopic shift, irregular astigmatism, and thinning cornea. Six eyes (12.8%) still showed progressed infection, and conjunctival flap covering, amniotic membrane transplantation, or penetrating keratoplasty were given. PTK is a valuable therapeutic alternative for superficial infectious keratitis. It can effectively eradicate lesions, hasten reepithelialization, and restore and preserve useful visual function. However, the selection of surgery candidates should be conducted carefully.

## 1. Introduction

Since the U.S. Food and Drug Administration approved the 193 nm argon-fluoride excimer laser for phototherapeutic keratectomy (PTK) procedures in 1995, a variety of anterior corneal pathologies have been treated by PTK with promising results [1]. Four broad categories of conditions that are candidates for PTK have been identified: anterior corneal scars, dystrophies of epithelium and Bowman's membrane, anterior corneal stromal dystrophies, and elevated corneal lesions [2]. Advantages of PTK include precise control of corneal ablation, ease of use, provision of a smooth base for corneal reepithelialization, relatively fast visual recovery, and the ability to repeat treatments. The applications for PTK, as well as our understanding of how it affects the biomechanical properties of the cornea, are still evolving.

Important updates and new usages of PTK have been reported recently in infectious keratitis. Many studies have reported successful PTK results in cases with various types of keratitis [3–6]. It is believed that the ablation and modeling effects of PTK could be used to eradicate infected tissue and organisms from the cornea and, at the same time, produce a more regular corneal surface after healing [7, 8].

In the present study, we retrospectively investigated the therapeutic safety and efficacy of excimer laser PTK for the treatment of 47 consecutive cases of patients with fungal keratitis of the anterior stroma.

## 2. Patients and Methods

### 2.1. Patients

We performed a retrospective study of patients newly diagnosed with fungal keratitis and treated by PTK. Consecutive cases at the inpatient clinic of the Department of Ophthalmology from January 2005 to September 2012 at No. 181 Hospital of PLA were investigated. All of the recruited patients were treated by a single surgeon (Liang Mao Li). Forty-seven eyes of 47 patients (19 female, 28 male) were included in the study. All patients had unilateral surgery. Patient age at the time of surgery ranged between 24 and 73 years (mean 51 years).

Written informed consent for the procedures was obtained from each patient. Institutional review board/ethics committee approval was obtained from No. 181 Hospital of PLA Institutional Review Board, and the study protocol followed the guidelines of the Declaration of Helsinki.

### 2.2. Diagnosis

Once a patient with suspected fungal keratitis presented at the clinic, cultures of the corneal scrapings and smear staining were performed before any medication was given. Sabouraud agar was used for cultures. Conventional bacterial culturing was also performed. At the initial visit, a detailed ophthalmic and medical history was taken, noting any history of trauma, predisposing factors, or family history of corneal diseases. The new diagnosis of fungal keratitis was based on clinical fungal manifestations seen on slit lamp examination as previously described [9] and/or previous identification of a pathogen on microscopic analysis (smears staining) or after culturing of the corneal scrapings. History of botanical trauma and ocular symptoms were additional references to the diagnosis of some cases. On confirming the diagnosis of fungal infection, the patients received combination treatment including topical or/and systemic antifungal medication, such as natamycin or amphotericin B. Topical antibacterial agents, such as levofloxacin and tobramycin, were used for prophylactic administration.

### 2.3. Inclusion and Exclusion Criteria

Inclusion criteria for this study were patients with fungal keratitis involving the anterior stroma. The infiltration depth or enlarged abscess was limited to a depth of less than half of the corneal thickness as determined by slit lamp microscopic examination. In most cases, the lesions were diffuse and limited to about one-third of the superficial corneal stromal layer. The residual corneal thickness without inflammatory edema after PTK was expected to be more than 300 *μ*m. The candidates for PTK surgery were cases in which the infection had no progression and had no apparent improvement regardless of combined medical therapy after at least one week. Improvement was defined as at least a 0.5 mm reduction in area or apparent reduction in depth of the infection infiltration occurring within seven days after treatment and was confirmed by a slit lamp or photographic examination. No hypopyon was present, or hypopyon had been resolved, after antimicrobial medications. The minimum follow-up period for inclusion was six months after surgery.

Exclusion criteria were immune compromised patients, patients with glaucoma, patients with severe blepharitis, or any condition that could significantly adversely affect corneal healing. One patient was excluded because he was lost to follow-up less than six months after PTK. One patient refused to accept the follow-up treatment for residual infection after PTK and was excluded.

### 2.4. Preoperative Evaluation

Characterizations of patients, including age, gender, cause, and associated conditions, were collected. The interval from the onset to the start of medical treatment and the interval from onset of therapy to PTK were also recorded. Preoperatively, all patients underwent a slit lamp examination, spherical equivalent measurement, keratometry, corneal topography (Orbscan II, Bausch & Lomb Inc., Rochester, NY, USA), uncorrected visual acuity exam, best spectacle-corrected visual acuity (BCVA) determination when possible, intraocular pressure measurement with Goldmann applanation tonometry, or noncontact tonometry (not possible to perform in all cases). Ultrasound pachymetry (pachymeter SP-3000, Tomey Laboratories, Inc., Phoenix, AZ, USA) was performed on all eyes preoperatively to assess suitability for PTK.

On the day of surgery, digital slit lamp photography was performed to determine the lesion size. Central corneal thickness (CCT), central lesion thickness, and peripheral lesion thickness measurements were taken. Accordingly, the thickness of corresponding areas on the nonaffected eye was also determined. The preoperative BCVA was the last recorded value before the excimer laser procedure was performed. The spherical equivalent and BCVA of the healthy eyes were also measured and recorded.

### 2.5. Surgery and Initial Postoperative Management

The surgical planning was based on Lin et al. study [5] and the experiences used in our center. The Technolas 217z excimer laser system (Bausch & Lomb Inc.) was used for all treatments. The laser system was an argon-fluoride 193 nm laser with a repetition rate of 50 Hz and a fluence of 120 mJ/cm^2^. After topical local anesthesia, an eyelid speculum was positioned. The standard surgery procedure consisted of the following steps.

1. The procedure in all patients began with manual debridement. The lesions, including epithelium and necrotic tissues, were manually scraped with a number 15 Beaver blade while being observed under the laser microscope, and samples were obtained for laboratory analysis (cultures, smear staining, and histopathology examination).
2. After collection of samples for microbial analysis, the ulcer was washed with povidone iodine (5%) and washed with Merocel sponges. Viscous sodium hyaluronate gel (1.0%) was applied to the peripheral ablation zone to protect the surrounding healthy tissue.
3. The diameter of initial ablation was determined so that the laser broad beam could just cover the entire lesion. The planned ablation depth was determined by taking the values of ultrasound pachymetry measurements (central corneal thickness, central lesion thickness, and peripheral lesion thickness) and the depth of the infiltrate. The postoperative residual corneal thickness without inflammatory edema was expected to be more than 300 *μ*m. Each laser ablation focus was the center of the lesion; therefore the ablation beam was aimed at the center of the lesion. The initial ablation depth was set at 25 *μ*m, and subsequent adequate ablative depth was determined by repeated assessment of clearing of the lesions between the laser bursts, visualized through the operating microscope. The small beam area was also used to ablate the deeper focal lesions. The end point was reached when the lesions were removed.
4. After completion of excimer laser ablation, the residual corneal stroma was thoroughly irrigated by balanced salt solution (BSS). Antimicrobial drops were instillated and a soft contact lens bandage (PureVision, Bausch & Lomb Inc.) was placed on the eye.
5. Oral pain medications were given as needed. The postoperative medication regimen generally consisted of topical antifungal and antibacterial drops, artificial tears, a topical cycloplegic, and systemic antimicrobial drugs. A contact lens (CL) was worn or repeatedly applied until the corneal epithelium was completely healed.

### 2.6. Follow-Up Examination

In the hospital, the patients were monitored daily for corneal reepithelialization, infiltration, and inflammation. After 2-3 days postoperatively, the CL was removed to check for reepithelialization. The CL was replaced with a new one on alternate days or each day until reepithelialization occurred, and the medication regimen was adjusted accordingly. If the infection worsened (i.e., the infiltration progressed, there was an increase in size of ulcer or infiltrate, or presence of perforation was detected) after PTK, aggressive surgery of conjunctival flap covering, amniotic membrane transplantation, or penetrating keratoplasty was performed.

Follow-up continued for at least six months. At the latest follow-up visit, the final visual acuity, refraction status, corneal clarity, and complications were noted. A slit lamp examination was conducted. Corneal haze was graded as follows: 0 for completely clear cornea; 0.5 for haze barely discernible in the slit lamp; 1 for faint haze detectable only with broad tangential illumination; 2 for discrete haze visible with difficulty by focal illumination, refraction possible; 3 for moderately dense opacity partially obscuring iris details; 4 for severely dense opacity completely obscuring details of intraocular structures [10].

## 3. Quantitative Analysis and Statistics

The results were presented as means and standard deviation (SD). SPSS software version 13.0 (SPSS, Inc., Chicago, IL, USA) was used for statistical analysis. The Wilcoxon signed-ranks test was used to evaluate the differences in pre- and postoperative BCVA. To elucidate the factors independently associated with surgery failure rate, we performed multivariate analyses of selected variables (age, gender, duration to surgery, diameter of the lesions, hypopyon once it appeared, size, and depth of ablation) by using binary logistic regression models with the stepwise method. *P* < 0.05 was considered to be statistically significant.

## 4. Results

### 4.1. Patient Characteristics

Forty-seven eyes of 47 patients who had undergone PTK were identified on a retrospective chart review during a seven-year period. The mean follow-up time was 11.7 ± 7.2 months (range 6 to 31 months).

The most common predisposing factor was a history of botanical trauma (30 eyes, 63.8%), mostly injured by branches, leaves, and vegetables. Eleven patients (23.4%) had experienced foreign body damage, such as sand or dust entering the eye. No notable risk factors were identified in six cases (12.8%).

Twenty-four patients (51.1%) did not receive immediate therapy, and the interval from the onset to the start of medical treatment was 3.1 ± 3.4 days (from 1 to 15 days). Twenty-five patients (53.2%) had experienced at least two treatment regimens at different hospitals or clinics, not including our hospital. The interval from onset of therapy to PTK was 31.2 ± 23.2 days (from 7 to 120 days) in all patients. Seventeen patients (36.2%) had a long treatment history of more than one month, and 33 patients (70.2%) developed corneal ulcers.

The diameter of the lesions was 3.89 ± 1.29 mm (from 1.5 to 7.0 mm). Hypopyon was examined in 22 eyes (46.8%) during medication treatment at our hospital or at other clinics.

Cultures and smear staining were performed twice for each eye before any medication at our hospital and before laser ablation. For each eye, histopathology examination was performed before laser ablation. The specimens were obtained by scraping the base and edges of the lesions. Some patients received medications before they came to our hospital, so the positive lesion rate was low in these patients. The results are shown in Table 1. Twenty cases (42.5%) had positive results. One case showed positive smear staining for fungi, 13 cases showed positive histopathology results for fungi, one case showed positive fungal cultures, and 11 cases showed positive bacterial cultures. Eleven cases (23.4%) had mixed infections, both bacterial and fungal.

### 4.2. Successful Cases

**Figure 1:**
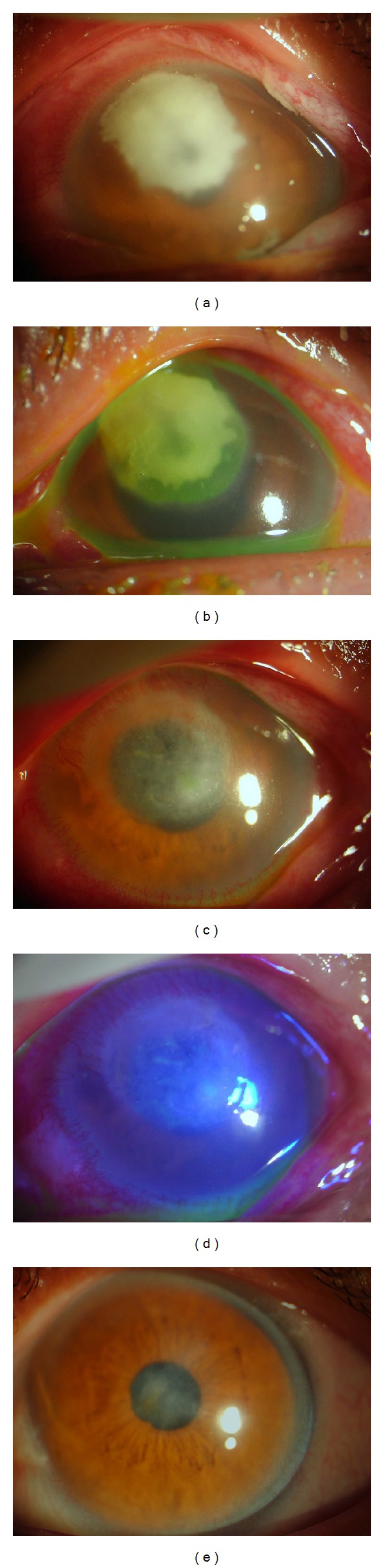
A patient with fungal keratitis. The patient's cornea is shown preoperatively with the lesions located in the anterior stroma (a) and by fluorescein staining (b). The cornea is shown at eight days postoperatively with phototherapeutic keratectomy (c) and with complete reepithelialization (d). The patient, at 18 months postoperatively, shows a smooth corneal surface with mild haze (e), and the best-corrected visual acuity is 0.5 (+2.50 DS  ∗ − 2.00 DC × 55°). The thinnest thickness of ablation zone is 472 *μ*m.

Forty-one patients (87.2%) treated with PTK were completely cured without recurrence during the follow-up period (Figure 1). The mean ablation depth was 114.39 ± 45.51 *μ*m (range 50 to 200 *μ*m) and diameter of ablation was 4.06 ± 1.07 mm (range 2.0 to 6.0 mm). Most patients experienced rapid reepithelialization in 8.8 ± 5.6 days (range 3 to 30 days). Five eyes showed delayed epithelial healing and required application of a bandage contact lens for a period of two weeks. The length of hospital stay ranged from 3 to 33 days (average 11.0 ± 8.1 days). Under slit lamp microscopic examination, the corneal epithelium showed progressive migration over the smooth ablation zone, without adhesive debris or necrotic tissue. After discharge from the hospital, corneal surface inflammation and infiltration were usually cured or remitted.

### 4.3. Visual Outcomes

In agreement with the improved clinical findings, the postoperative BCVA was significantly better than the preoperative BCVA (*Z* = −5.454, *P* < 0.01) (Table 2). None of the patients had lost a line of BCVA. A total of seven eyes (17.1%) were within one line of their preoperative BCVA. Thirty-four eyes (82.9%) showed an improvement in BCVA of 2 or more lines, four eyes gained 3 lines, 10 eyes gained 4 lines, six eyes gained 5 lines, two eyes gained 6 lines, two eyes gained 7 lines, three eyes gained 8 lines, one eye gained 9 lines, one eye gained 10 lines, and three eyes gained 11 lines.

### 4.4. Haze Results

The mean postoperative corneal haze grading was 2.78 ± 0.82 (range 1 to 4). Twenty-six eyes (55.3%) had a haze grading of 3 or greater. Most of the haze (in 31 eyes, 65.9%) was localized in the pupil area of the cornea and apparently influenced the visual axis.

### 4.5. Refractive Change

The pre-PTK refraction data was not available in some infectious lesion cases and some cases postoperatively showed irregular astigmatism. Therefore, it was not possible to report the amount of hyperopic shift and astigmatism in these cases. The remaining eyes showed hyperopic shift postoperatively from +0.50 to +2.75 D and astigmatism from 0 to −3.75 D.

### 4.6. Corneal Thickness

In addition to the infection recurrence after PTK, corneal thinning was a major complication. Of all patients' residual stromal bed, the thinnest portion of the ablation zone was 385 *μ*m, and there was no trend of ectasia.

### 4.7. Failure Cases

Infections still progressed in six cases (12.8%), after PTK, which had undergone other surgical treatments. The findings of these unsuccessful cases are summarized in Table 3.

On multivariate analyses, diameters of the lesions (odds ratio (OR) = 7.329; 95% confidence intervals (CI) = 1.496−35.897; *P* = 0.014) and size of ablation (OR = 5.161; 95% CI = 1.305−20.414; *P* = 0.019) were the factors that were independently associated with the failure rates. The patients' ages (OR = 0.979; 95% CI = 0.912−1.051; *P* = 0.553), gender (OR = 1.562; 95% CI = 0.280−8.716; *P* = 0.611), hypopyon (OR = 1.158; 95% CI = 0.209−6.428; *P* = 0.867), duration to surgery (OR = 1.029; 95% CI = 0.996−1.064; *P* = 0.090), and depth of ablation (OR = 1.008; 95% CI = 0.991−1.025; *P* = 0.381) were not the independent factors associated with the failure rate.

## 5. Discussion

Infectious keratitis is a well-recognized cause of visual loss or blindness worldwide [11]. In the developing world, agricultural work and outdoor occupations appear to predispose to infectious keratitis, and fungi are the common pathogen for cornea infection [12].

Infectious keratitis is an ophthalmologic emergency. Thus the immediate initiation of an appropriate and aggressive therapy is needed to halt the disease process and limit the extent of corneal scarring and loss of vision. Most of the patients in the present study lived in mountainous areas with poor economic development and inconvenient travel. Therefore, they sometimes did not receive effective treatment or even underwent inappropriate treatment. The fungal infectious lesions were prone to develop into dormant ulcers, resistant to antimicrobial or combined medical treatment, while mixed infections were prone to a worse prognosis.

Out of 653 cases with infectious keratitis in the last seven years in our hospital, 47 eyes with infectious lesions, located in the anterior stroma, were selected for PTK. It was important to carefully determine the candidates for surgery. Besides the inclusion and exclusion criteria listed in Section 2, corneas with as little edema as possible, were selected. Because the boundary between the diffuse edema and the infection infiltration was not clear, it was difficult to determine whether the lesions were completely removed by laser ablation. More importantly, candidate surgery cases were determined by an explicit percentage of infiltration.

It was also difficult to determine the depth of infectious lesions. Less infection depth means less ablation depth, and this factor could possibly reduce the risk of unpredictable perforation or ectasia. Owing to a lack of an Anterior Segment Optical Coherence Tomography instrument, or Pentacam high-resolution rotating Scheimpflug imaging system, we only used the slit lamp biomicroscopy to carefully observe and roughly estimate lesions. The thickness of the ulcerative and peripheral healthy portions around the lesions was measured by ultrasound pachymetry. The thickness of the contralateral healthy eye was also measured as control. Subtracting the missing thickness due to the ulcer and the infiltration thickness, the residual stroma bed thickness, after ablation, was expected to be more than 300 *μ*m without edema. When necessary, in severe cases, we used ultrasound pachymetry to measure the residual stromal bed thickness, before laser ablation, thus avoiding excessive ablation.

We did not directly use a masking agent, because, as previously reported by others [5], our priority was to eradicate the infection. This factor may have contributed to patient astigmatism. Masking agents on the corneal surface may disturb the precision of ablation of the focal lesion, and this could result in ablation of more normal tissue. Furthermore, a masking agent would make the calculation of ablation depth more difficult [5]. In our study, we applied viscous sodium hyaluronate (1.0%) to the peripheral ablation zone to protect the surrounding healthy tissues. During laser ablation, the cleared zone appeared from the peripheral lesion zone to the center. According to our observations and owing to the viscosity and fluidity of sodium hyaluronate, it first flowed slowly from the healthy tissues to the peripherally cleaned lesion zones. Therefore, sodium hyaluronate appeared to indirectly act as a masking agent, but it did not impede excimer laser ablation to eliminate lesions in the early stage. The sodium hyaluronate also helped in achieving a smooth surface in the later stage of ablation.

In the 41 successful cases, the mean time for healing of the epithelial defect was 8.8 ± 5.6 days and the mean length of hospital stay was 11.0 ± 8.1 days. Lin et al. compared 9 patients with superficial keratomycosis that received PTK to 31 cases of keratomycosis that had been treated with traditional surgical procedures and topical antifungal agents [5]. They revealed that much less time was needed to treat the PTK group (12.9 ± 3.6 days) than the control group (40.8 ± 26.4 days), and the PTK group experienced rapid reepithelialization (3.6 ± 1.8 days).

Although apparent improvement in BCVA occurred in successful cases after PTK, the prognosis was given based on the lesion location, size, and depth. The Lin et al. study showed that the PTK group had an average visual improvement of 2.9 ± 2.1 lines, which was significantly better than the final improvement found in the control group (0.6 ± 1.7 lines) [5]. Hyperopic shift and astigmatism were sometimes observed. The changes in refraction would be expected to be significant but were not extensively tested owing to there being minimal preinfection data available. Wavefront-guided or topography-guided customized refractive laser treatment could be performed in the future, if needed. Haze normally accompanied PTK but should be much less severe than scarring, but only after natural healing or manual debridement. This should be verified by a further comparative study. Both haze severity and location influenced BCVA improvement, but a correlation study between haze severity and visual acuity was not performed.

Corneal thinning or iatrogenic keratectasia should also be considered [1, 13]. Preoperative stringent evaluation, intraoperative application of ultrasound pachymetry, and postoperative close follow-up should effectively prevent this major complication. Additionally, the ablation diameter was the same as the lesion diameter or slightly greater. The lesions were only just covered by the optical zones. These could avoid excessive operative trauma on healthy corneal tissue. Sufficient residual healthy corneal tissue is needed to prevent keratectasia or perforation.

There were only 13 laboratory confirmed fungal keratitis patients in the 47 cases. One reason was the limitation of the laboratory conditions and experiences. Another was possibly the effect of antifungal drops before pathogen examination; these lesions became stationary and refractory. In this study, infection progression remained a severe complication after PTK. Among the 6 failed PTK treatment patients, 5 cases were confirmed by laboratory examination. It was possibly inferred that there were active lesions in these failure cases.* Aspergillus* and* Candida* species were identified as pathogens in the failure cases. Experimental and clinical studies previously reported different growth patterns and clinical therapeutic effects [14] of fungal hyphae in corneas. Most fungal hyphae of* Aspergillus* and* Candida* species grow vertically in corneal stroma and easily invade the deep stroma, even penetrating Descemet's membrane and into the anterior chamber. Horizontally growing hyphae such as* Fusarium* may also invade the deep corneal lamellae during the progression of fungal infection. For cases of lamellar keratoplasty, even if the lesions were completely cleaned, which were evidenced by KOH staining intraoperatively, the rudimentary fungi in the deep stroma still caused fungal recurrence postoperatively [15, 16]. As shown by our study, PTK did not thoroughly eradicate the latent fungi because it was necessary to reserve sufficient corneal stroma. Although multivariate analysis results did not verify that duration from onset to surgery was a risk factor independently associated with the failure rate, for patients unresponsive to antifungal agents, PTK still should be employed as early as possible. On the other hand, PTK might not be suitable for active lesions of fungal keratitis. This finding underscored the importance of surgical timing in the treatment of superficial fungal keratitis by PTK.

The multivariate analysis results indicated that diameters of the lesions and sizes of ablation were risk factors independently associated with failure rate. These indicators reflected the severity of infection. The option of PTK for these cases is to be cautioned. Because of the corneal ulcer and manual debridement before PTK, depth of ablation could not reflect the depth of lesions and the multivariate analysis showed no statistical significance.

The multivariate analysis results revealed that hypopyon was not an independent factor associated with failure rate, but the presence of hypopyon should be noted before consideration of PTK. Obviously, for infectious keratitis, the phenomenon of hypopyon suggested that the infection possibly progressed into the anterior chamber and deep stroma, which were invaded by pathogens. Even if hypopyon was resolved by medications, fungi could still be latent and dormant, in the deep stroma. In the future, a larger study sample size is needed to verify whether hypopyon is an independent factor associated with failure rate.

In summary, excimer laser PTK is an effective tool for the management of superficial fungal keratitis refractory to conventional therapy. It is a minimally invasive procedure that is often successful in delaying or avoiding more aggressive corneal surgeries, such as lamellar or penetrating keratoplasty. In contrast to simple surgical debridement, this method can precisely remove stromal lesions, provide for a smooth and clean corneal surface, hasten reepithelialization, shorten the duration of treatment, and restore reasonably good vision. However, the cost-effectiveness of PTK should also be a concern, especially for a developing country.

PTK may provide a possible treatment for the management of superficial fungal keratitis, especially when the fungi are still latent in the deep stroma. In developing countries, lamellar keratoplasty or penetrating keratoplasty may not be an option for deep stromal invasion owing to lack of donor corneas. However, for keratitis patients who fail to respond to medical therapy, PTK is one option. It may be simultaneously combined with intracameral and intrastromal antimycotic agents, conjunctival flap covering, or amniotic membrane transplantation. In this manner, corneal perforation could be avoided, and the progression of infection could be delayed. Based on the results of our study, PTK should be investigated for use as one treatment for nonresponding cases. In the future, long-term and comparative studies are required to evaluate more completely the efficacy of PTK to treat superficial fungal keratitis.

## Figures and Tables

**Table 1:** Results of pathogen examination from 47 clinically presumed fungal keratitis subjects.

Case	Laboratory examination		
	Smear staining	Culture	Histopathology
1	−	*Staphylococcus epidermidis *	*Aspergillus *
2	−	−	Fungi
3	−	−	*Candida *
4	*Aspergillus *	−	*Aspergillus *
5	−	*Staphylococcus aureus *	−
6	−	−	*Candida *
7	−	−	Fungi
8	−	*Staphylococcus aureus *	−
9	−	Hemolytic *Staphylococcus *	−
10	−	*Aspergillus flavus *	Fungi
11	−	−	Fungi
12	−	Hemolytic *Streptococcus *	Fungi
13	−	*Escherichia coli *	−
14	−	−	Fungi
15	−	−	Fungi
16	−	*Staphylococcus epidermidis *	−
17	−	*Pseudomonas alcaligenes *	Fungi
18	−	*Staphylococcus epidermidis *	−
19	−	*Staphylococcus epidermidis *	Fungi
20	−	*Streptococcus pneumoniae *	−
21–47	−	−	−

Total	1	12	13

−: negative.

**Table 2:** Changes of best-corrected visual acuity for 41 successful cases.

Visual acuity	Preoperative	Postoperative
1.0–0.5	3	14
0.4–0.2	4	13
0.15–0.05	8	12
Below 0.05	26	2

**Table 3:** Demographic, clinical, and surgical data on six failure cases with phototherapeutic keratectomy.

Case	Gender/age (y)	Predisposing factors	Pathogen	Original findings at our hospital	Duration to PTK (d)	Ablation data	Evolution
1	F/44 LE	Botanical trauma	His: *Aspergillus* Culture: *S. epidermidis *	Ulcer: 5.0 × 6.0 mm Hypopyon: 3 mm VA: HM	52	Size: 6.0 mm Depth: 75 *μ*m	Infection progression, AMT at 13 days

2	M/60 RE	Sand entering the eye	His: fungi	Ulcer: 5.0 × 4.0 mm Hypopyon: (−) VA: CF/20 cm	26	Size: 5.0 mm Depth: 50 *μ*m	Infection progression, AMT at 12 days

3	M/43 LE	Botanical trauma	His: *Candida *	Ulcer: 4.5 × 4.0 mm Hypopyon: 2 mm VA: HM	73	Size: 4.0 mm Depth: 75 *μ*m	Infection progression, AMT at 18 days

4	M/44 RE	Botanical trauma	Smear: *Aspergillus* His: *Aspergillus *	Ulcer: 7.0 × 7.0 mm Hypopyon: (−) VA: HM	45	Size: 6.0 mm Depth: 200 *μ*m	Infection progression, CFC at 2 days

5	M/53 LE	Botanical trauma	Culture: *S. aureus *	Ulcer: 4.0 × 6.0 mm Hypopyon: (−) VA: CF/10 cm	70	Size: 6.0 mm Depth: 200 *μ*m	Infection progression, CFC at 13 days

6	F/47 RE	Botanical trauma	His: *Candida *	Ulcer: 4.8 × 4.0 mm Hypopyon: 2 mm VA: CF/20 cm	28	Size: 5.0 mm Depth: 200 *μ*m	Infection progression and perforation, PK at 9 days

F: female, M: male, LE: left eye, RE: right eye, y: year, d: day, VA: visual acuity, HM: hand motion, CF: count finger, CFC: flap covering, AMT: amniotic membrane transplantation, PK: penetrating keratoplasty, His: histopathology examination, *S. epidermidis: Staphylococcus  epidermidis, and S. aureus: Staphylococcus  aureus*.
